# Low-dose vs. standard-dose alteplase for acute ischemic stroke: a real-world single-center retrospective study

**DOI:** 10.3389/fneur.2025.1651307

**Published:** 2025-10-21

**Authors:** Xin-Lei Mao, Si-Si He, Ya-Xi Zhang, Cai-Dan Lin, Xin-Xin Chen, Shi-Zheng Zhang, Li-Na Ge, Qing-Qing Zhuang

**Affiliations:** Department of Neurology, Wenzhou Central Hospital & Dingli Clinical Institute of Wenzhou Medical University, Wenzhou, Zhejiang, China

**Keywords:** acute ischemic stroke, alteplase, low-dose, standard-dose, real-world study

## Abstract

**Objectives:**

To investigate the real-world usage patterns and clinical outcomes of low-dose (0.6 mg/kg) versus standard-dose (0.9 mg/kg) intravenous alteplase in patients with acute ischemic stroke (AIS).

**Methods:**

This single-center retrospective study included 707 patients with AIS who received intravenous thrombolysis at Wenzhou Central Hospital from December 2016 to April 2023. Baseline characteristics, treatment selection, and clinical outcomes were analyzed. The primary outcomes were 90-day functional outcomes (mRS 0–1 and mRS 0–2) and the incidence of symptomatic intracranial hemorrhage (sICH).

**Results:**

Low-dose alteplase was more commonly used in older patients (*p* < 0.001) and in those on pre-stroke antithrombotic medications (*p* < 0.01). Junior physicians were more inclined to use low-dose alteplase compared to senior physicians (*p* < 0.001). The coefficient of variation in rt-PA dosage selection among different physicians was as high as 61%. There were no significant differences in 90-day functional outcomes (mRS 0–1: OR 0.87, 95% CI 0.62–1.23, *p* = 0.43; mRS 0–2: OR 1.09, 95% CI 0.78–1.52, *p* = 0.63) or the incidence of sICH (by NINDS criteria: OR 1.68, 95% CI 0.78–3.62, *p* = 0.19) between the low-dose and standard-dose groups.

**Conclusion:**

The study highlights the complexity of treatment decision-making for intravenous alteplase in AIS, with significant influences from both patient and physician factors. Low-dose alteplase demonstrated similar clinical outcomes to standard-dose alteplase in this real-world setting. Future research should focus on optimizing treatment decisions and improving guideline adherence to enhance patient outcomes.

## Introduction

Acute ischemic stroke (AIS) is a leading global cause of disability and mortality. Intravenous thrombolysis (IVT) is the primary treatment for AIS, and current guidelines recommend the use of standard-dose (0.9 mg/kg) recombinant tissue plasminogen activator (rt-PA) for treatment (1–3). Nevertheless, the standard dose is associated with an elevated risk of symptomatic intracranial hemorrhage (sICH), potentially compromising clinical outcomes. In recent years, low-dose (0.6 mg/kg) rt-PA has garnered widespread attention as an alternative treatment option, especially in Asian regions (4–6). Whether low-dose rt-PA achieves comparable efficacy to the standard dose while mitigating sICH risk has been widely debated. Although previous studies have explored the efficacy and safety of low-dose versus standard-dose rt-PA (7, 8), data from real-world settings remain limited. This study aims to collect real-world clinical data to analyze real-world usage patterns and clinical outcomes of low-dose versus standard-dose rt-PA in AIS, investigate the influence of patient- and physician-related factors on treatment selection, and explore the underlying decision-making influences driving these choices.

## Materials and methods

### Study population

This single-center, retrospective, observational study included consecutive patients with acute ischemic stroke (AIS) who received intravenous thrombolysis with rt-PA at Wenzhou Central Hospital between December 2016 and April 2023. A total of 707 patients were enrolled: 192 received low-dose rt-PA and 515 received standard-dose rt-PA. Inclusion criteria were as follows: (1) age ≥18 years, (2) symptoms of neurological deficits, with a definitive diagnosis of AIS confirmed by cranial magnetic resonance imaging (MRI) or computed tomography (CT) imaging, (3) treatment initiation within 4.5 h of symptom onset (or last-known-well time). Exclusion criteria included: (1) lack of documented informed consent, (2) presence of contraindications to intravenous thrombolysis, (3) treatment with other thrombolytic agents, (4) receipt of arterial bridging therapy.

### Data collection and assessment

Baseline demographic and clinical characteristics of the patients were collected, including age, sex, body mass index (BMI), past medical history (such as hypertension, diabetes, atrial fibrillation, etc.), stroke subtype (TOAST classification), Admission National Institutes of Health Stroke Scale (NIHSS) score, blood glucose level, systolic and diastolic blood pressure, etc. Additionally, door-to-needle time (DNT) and the modified Rankin Scale (mRS) score at 3 months were recorded. Furthermore, characteristics of the treating physicians were collected, including sex, seniority (all physicians were certified neurologists with master’s or doctoral degrees; defined by the number of years of clinical practice after completing residency training, with senior physicians ≥5 years and junior physicians <5 years), the usage rate of low-dose rt-PA, and thrombolysis refusal rate within the 4.5-h time window (excluding contraindications).

### Statistical analysis

Statistical analysis was performed using SPSS Statistics version 26.0 (IBM Corporation, Armonk, NY, United States). Descriptive statistics were used to analyze the baseline characteristics of the patients. Chi-square tests and t-tests were employed to compare differences between the low-dose and standard-dose groups. The variations in rt-PA dosing practices among clinicians were analyzed. Binary logistic regression analysis was used to adjust for confounding factors and to assess the impact of low-dose versus standard-dose rt-PA on clinical outcomes, including 90-day functional outcomes (mRS 0–1 and 0–2) and the incidence of sICH.

## Results

**Figure 1:**
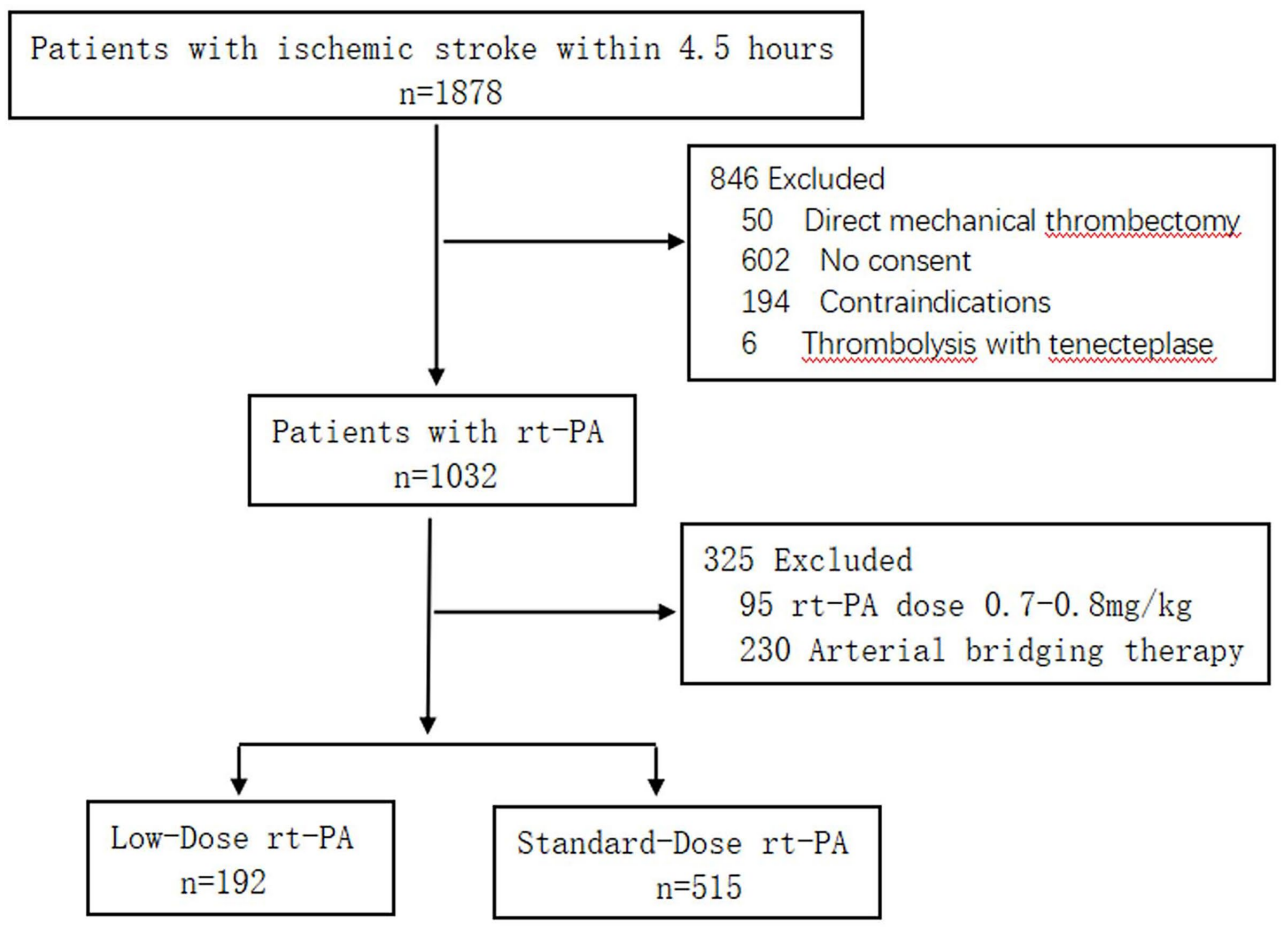
Flow diagram of the study.

The final study population consisted of 707 patients, as shown in Figure 1. The baseline characteristics of patients in the low-dose group (*n* = 192) and the standard-dose group (*n* = 515) are presented in Table 1. Compared with the standard-dose group, the low-dose group had a significantly higher mean age (*p* < 0.001) and a significantly higher rate of antiplatelet or anticoagulant use before thrombolysis (*p* < 0.01). There were no statistically significant differences between the two groups in terms of sex, BMI, past medical history (such as hypertension, diabetes, atrial fibrillation, etc.), stroke subtype, Admission NIHSS score, blood glucose level, systolic and diastolic blood pressure, etc. The median DNT was 54 min in the low-dose group, which was significantly higher than the 47 min in the standard-dose group (*p* < 0.001).

**Table 1:** Baseline demographics and clinical characteristics of the patients.

Characteristics	0.6 mg/kg (*n* = 192)	0.9 mg/kg (*n* = 515)	*p* value
Age, years (mean, SD)	75.09 ± 13.13	67.77 ± 13.58	0.00
Male, *n* (%)	109 (56.77)	327 (63.50)	0.10
BMI (mean, SD)	23.75 ± 3.46	23.70 ± 3.69	0.90
Hypertension, *n* (%)	143 (74.48)	365 (70.87)	0.34
Diabetes mellitus, *n* (%)	66 (34.38)	168 (32.62)	0.66
Atrial fibrillation, *n* (%)	49 (25.52)	102 (19.81)	0.10
History of stroke, *n* (%)	24 (12.5)	63 (12.23)	0.92
Hyperlipidemia, *n* (%)	51 (26.56)	128 (24.85)	0.64
Coronary artery disease, *n* (%)	14 (7.29)	25 (4.85)	0.21
Smoking, *n* (%)	30 (15.63)	109 (21.17)	0.10
Alcoholism, *n* (%)	26 (13.54)	80 (15.53)	0.51
Pre-stroke antithrombotic medications			0.01
Antiplatelet, *n* (%)	21 (10.94)	35 (6.80)	
Anticoagulation, *n* (%)	8 (4.17)	8 (1.55)	
NIHSS, median (IQR)	5 (3–10)	6 (3–11)	0.28
ONT, median (IQR)	168 (119–220)	161.5 (118–206)	0.17
DNT, median (IQR)	54 (41–70)	47 (36–59)	0.00
Glucose (mmol/L), median (IQR)	7.6 (6.40–10.43)	7.8 (6.60–10.10)	0.73
Systolic blood pressure (mmhg) (mean, SD)	166.86 ± 26.30	167.01 ± 27.45	0.96
Diastolic blood pressure (mmhg) (mean, SD)	87.71 ± 18.69	90.94 ± 16.09	0.46
Stroke subtype, *n* (%)			0.48
Large artery atherosclerosis	40 (20.83)	113 (21.94)	
Small vessel occlusion	37 (19.27)	112 (21.75)	
Cardioembolism	42 (21.88)	87 (16.89)	
Other/Undetermined	73 (38.02)	203 (39.42)	
In-hospital mortality, *n* (%)			
Death from cardiovascular causes	8 (4.17)	21 (4.08)	0.96
Death from any cause	10 (5.21)	24 (4.66)	0.76

BMI, body mass index; ONT, onset-to-needle time; DNT, door-to-needle time; NIHSS, National Institutes of Health Stroke Scale; IQR, interquartile range.

Twenty-two physicians who had performed thrombolysis in more than 5 patients were included. There were significant differences in rt-PA dose selection among different physicians (Table 2). Physician 1 did not use low-dose rt-PA, while Physician 22 had the highest proportion of low-dose usage (81.82%) (*p* < 0.001), with a coefficient of variation (CV) as high as 61%. Junior physicians were more inclined to use low-dose rt-PA, whereas senior physicians were more inclined to use standard-dose rt-PA (*p* < 0.001).

**Table 2:** Interphysician agreement on the use of rt-PA dosing.

Characteristics	0.6 mg/kg (*n* = 187)	0.9 mg/kg (*n* = 497)	*χ^2^*	*P* value
Physicians			84.29	0.00
1	0	9		
2	1	33		
3	1	28		
4	1	15		
5	1	12		
6	8	50		
7	4	23		
8	5	25		
9	3	13		
10	3	11		
11	19	53		
12	21	58		
13	4	11		
14	22	42		
15	17	30		
16	10	17		
17	15	20		
18	29	32		
19	4	4		
20	4	4		
21	6	5		
22	9	2		
Sex			0.40	0.53
Male	73	181		
Female	114	316		
Experience stratification			26.13	0.00
Senior	72	300		
Junior	115	197		

**Figure 2:**
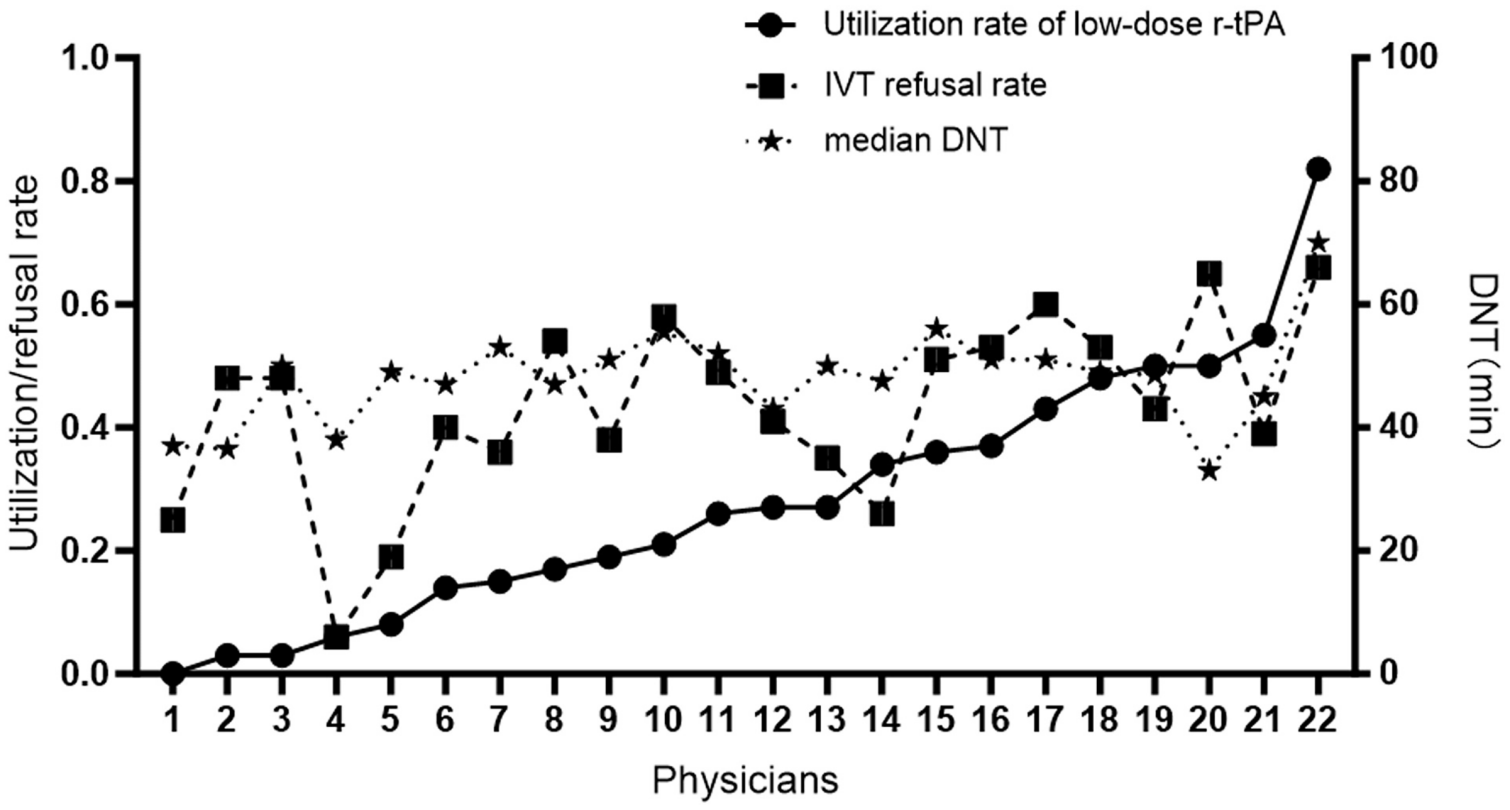
The correlation of low-dose rt-PA use rate with IVT refusal rate and median DNT.

There was a positive correlation between the usage rate of low-dose rt-PA and the refusal rate of IVT (r = 0.557, *p* = 0.007), after adjusting for seniority, the correlation remained significant (r = 0.69, *p* = 0.0005). A positive correlation was also observed between the usage rate of low-dose rt-PA and the median DNT (r = 0.46, *p* = 0.032), after adjusting for seniority, this correlation was further strengthened (r = 0.53, *p* = 0.015) (see Figure 2).

**Figure 3:**
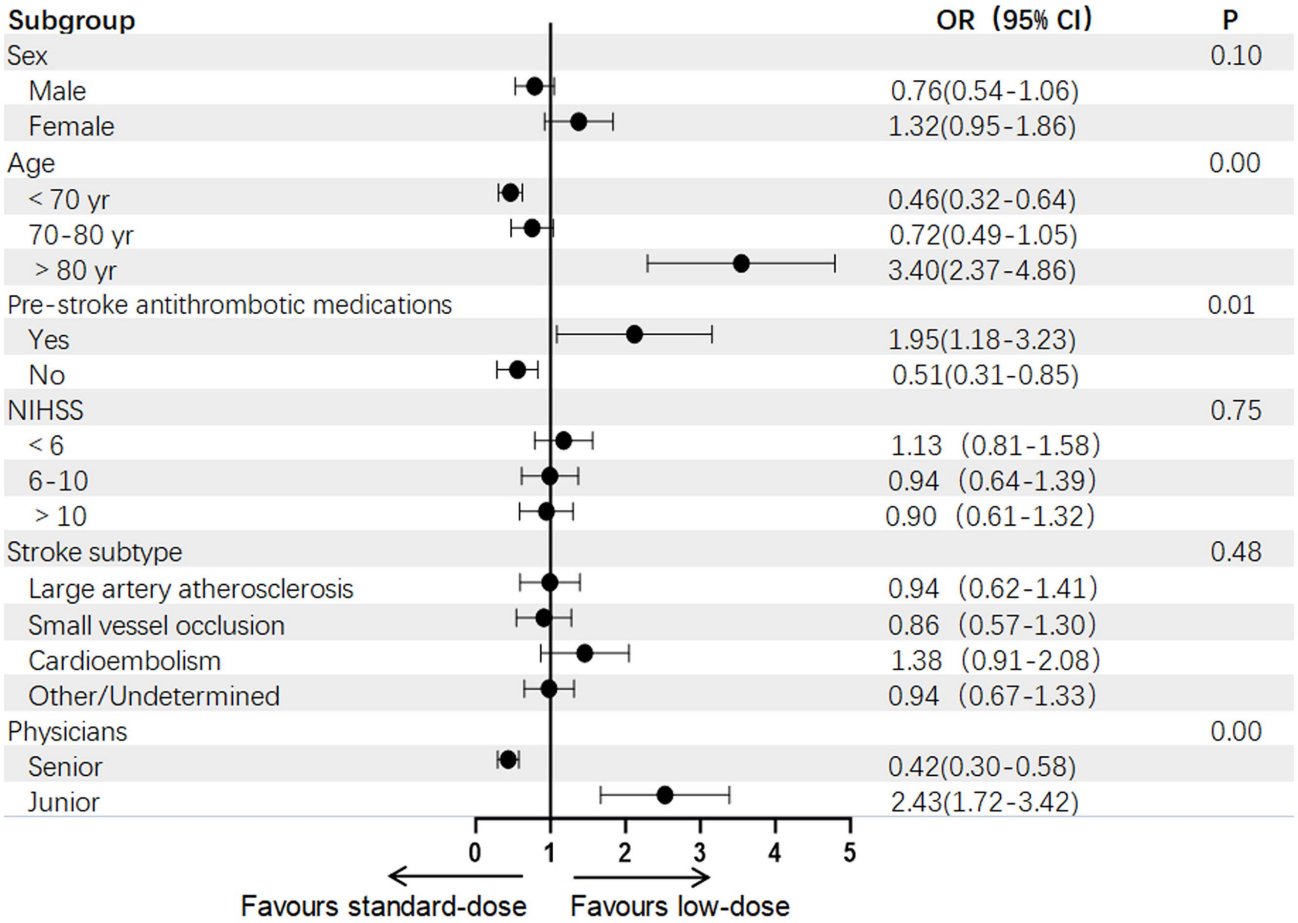
Subgroup analyses between the low-dose and standard-dose groups.

Figure 3 shows the impact of patient sex, age, pre-stroke use of antithrombotic medication, stroke severity, stroke subtype, and physician seniority on the selection of rt-PA dosage. When patients are over 80 years old or have been taking antithrombotic medication before the stroke, physicians tend to use a lower dose of rt-PA. Among physicians, junior physicians are more inclined to use a lower dose of rt-PA.

The 90-day functional outcomes and the incidence of ICH for the low-dose group and the standard-dose group are shown in Table 3. There was no significant difference in the proportion of mRS scores 0–1 and 0–2 between the low-dose group and the standard-dose group (*p* > 0.05 for both). There was also no significant difference in the incidence of any ICH and sICH between the two groups (*p* > 0.05 for both).

**Table 3:** Association between low-dose and standard-dose rt-PA and clinical outcomes.

Characteristics	0.6 mg/kg, *n* (%)	0.9 mg/kg, *n* (%)	Unadjusted OR (95%CI)	*P* value	Model 1 OR (95%CI)	*P* value	Model 2 OR (95%CI)	*P* value
mRS of 0–1	68/192 (35.42)	199/515 (38.64)	0.87 (0.62–1.23)	0.43	0.99 (0.68–1.44)	0.95	1.04 (0.72–1.49)	0.84
mRS of 0–2	115/192 (59.90)	298/515 (57.86)	1.09 (0.78–1.52)	0.63	1.21 (0.84–1.75)	0.31	1.38 (0.96–1.97)	0.08
SICH at 24–36 h								
By NINDS standard	11/192 (5.73)	18/515 (3.50)	1.68 (0.78–3.62)	0.19	1.34 (0.58–3.13)	0.49	1.33 (0.60–2.95)	0.49
By ECASS II standard	8/192 (4.17)	9/515 (1.75)	2.44 (0.93–6.43)	0.07	2.36 (0.78–7.20)	0.13	1.77 (0.66–4.80)	0.26
By SITS-MOST standard	7/192 (3.65)	7/515 (1.36)	2.75 (0.95–7.93)	0.06	2.67 (0.79–9.07)	0.12	2.09 (0.70–6.26)	0.19
Any ICH	29/192 (15.10)	65/515 (12.62)	1.23 (0.77–1.98)	0.39	1.18 (0.70–1.99)	0.54	0.95 (0.58–1.56)	0.85

mRS, modified Rankin Scale; ICH, intracerebral hemorrhage;sICH, symptomatic ICH; NINDS: National Institute of Neurological Disorders and Stroke; ECASS, European Cooperative Acute Stroke Study; SITS-MOST, Safe Implementation of Thrombolysis in Stroke-Monitoring Study. The model 1 adjusted for physicians. The model 2 adjusted for patients’ age and antithrombotic medication before the stroke.

Table 4 details antiplatelet and anticoagulant exposure prior to thrombolysis; Table 5 presents the baseline demographics and clinical profile of the SICH cohort.

**Table 4:** Antiplatelet and anticoagulant medications used before thrombolysis.

Therapy	0.6 mg/kg			0.9 mg/kg		
	*n*	Daily dose	Last intake time	*n*	Daily dose	Last intake time
Antiplatelet	21			35		
Aspirin alone	11	100 mg	≤24 h	23	100 mg	≤24 h (21 cases) 24-48 h (1 case) 48-72 h (1 case)
Clopidogrel alone	6	75 mg	≤24 h	8	75 mg	≤24 h
Aspirin+Clopidogrel	4	100 mg + 75 mg	≤24 h (3 cases) 48-72 h (1 case)	3	100 mg + 75 mg	≤24 h
Aspirin+Ticagrelor	-	-	-	1	100 mg + 180 mg	≤24 h
Anticoagulation	8			8		
Warfarin	4	2.5 mg (2 cases) 1.25 mg (2 cases)	≤24 h (3 cases) 48-72 h (1 case)	4	2 cases 1.88 mg 1 case 2.5 mg 1 case 0.83 mg	≤24 h
Rivaroxaban	3	10 mg (2 cases) 5 mg (1 case)	≤24 h (1 case) 48-72 h (2 cases)	3	10 mg	≤24 h (2 cases) 48-72 h (1 case)
Dabigatran	1	220 mg	≤24 h	-	-	-
Edoxaban	-	-	-	1	30 mg	48-72 h

**Table 5:** Baseline demographics and clinical characteristics of the SICH patients.

Characteristics	0.6 mg/kg	0.9 mg/kg	*P* value
	(*n* = 11)	(*n* = 18)	
Age, years (mean, SD)	80.18 ± 14.10	71.72 ± 12.07	0.10
Male, *n* (%)	6	10	0.96
BMI (mean, SD)	24.23 ± 2.69	26.03 ± 4.31	0.37
Hypertension, *n* (%)	8	13	0.98
Diabetes mellitus, *n* (%)	4	7	0.89
Atrial Fibrillation, *n* (%)	2	5	0.56
History of stroke, *n* (%)	0	4	0.09
Hyperlipidemia, *n* (%)	3	3	0.49
Coronary artery disease, *n* (%)	1	1	0.72
Smoking, *n* (%)	1	3	0.57
Alcoholism, *n* (%)	1	2	0.86
Pre-stroke antithrombotic medications			0.79
Antiplatelet, *n* (%)	1	3	
Anticoagulation, *n* (%)	1	1	
NIHSS, median (IQR)	13 (4.5–22)	5.5 (3–12)	0.13
ONT, median (IQR)	165 (130.75–185)	160 (111–219)	0.79
DNT, median (IQR)	66 (61.25–99)	58 (50–73.75)	0.23
Glucose (mmol/L), median (IQR)	7.45 (6.55–10.40)	9.1 (7.28–12.28)	0.25
Systolic blood pressure (mmhg) (mean, SD)	160 ± 13.44	161.21 ± 16.44	0.86
Diastolic blood pressure (mmhg) (mean, SD)	76 ± 10.80	85.79 ± 13.96	0.10
Stroke subtype, *n* (%)			0.39
Large artery atherosclerosis	0	3	
Small vessel occlusion	0	1	
Cardioembolism	4	4	
Other/Undetermined	7	10	
In-hospital mortality, *n* (%)			
Death from cardiovascular causes	6	6	0.26
Death from any cause	6	6	0.26

BMI, body mass index; ONT, onset-to-needle time; DNT, door-to-needle time; NIHSS, National Institutes of Health Stroke Scale; IQR, interquartile range. SICH was defined according to the NINDS standard.

## Discussion

This study, through the analysis of real-world clinical data, uncovers the current usage patterns of different doses of rt-PA in patients with AIS. Despite guidelines recommending the use of standard-dose rt-PA, the use of low-dose is relatively common among some physicians. This may reflect an awareness of the potential benefits of low-dose rt-PA, as well as considerations to reduce the risk some physicians. This may reflect an awareness of the potential of sICH. However, there are significant differences in this usage pattern among different physicians, suggesting that there may be multiple influencing factors.

Patient factors play a significant role in the decision-making process for the use of low-dose rt-PA. This study found that patients who are older and those who were on anticoagulant medication prior to the stroke are more likely to be treated with a lower dose. This may be because physicians consider these patients to have a higher overall risk, and low-dose rt-PA might offer a more balanced risk–benefit ratio (6). The significantly longer DNT in the low-dose group compared to the standard-dose group may suggest that patients in the low-dose group have more complex conditions (9). Additionally, patients’ clinical characteristics, such as stroke severity (NIHSS score) and blood Pressure levels, may also influence the physician’s choice (10–12).

This study indicates that physicians with less seniority are more inclined to use low-dose rt-PA, suggesting that a doctor’s treatment experience significantly influences the use of low-dose rt-PA. There is a positive correlation between the usage rate of low-dose rt-PA and the IVT refusal rate. This implies that when a physician is more inclined to use low-dose rt-PA, they are also more likely to refuse IVT. This could be due to inexperience, concerns about treatment risks, or other factors (13, 14). Furthermore, the coefficient of variation in rt-PA dosage selection among different physicians is as high as 61%, reflecting the differences in their understanding and adherence to treatment guidelines (15).

In terms of clinical outcomes, there was no significant difference between the low-dose and standard-dose rt-PA treatment groups regarding the 90-day functional outcomes (mRS 0–1 and 0–2) and the incidence of sICH. This suggests that the low-dose group had similarly favorable functional outcomes compared to the standard-dose group. Regarding safety, large-scale clinical studies have shown that the risk of hemorrhagic transformation increases with higher doses of rt-PA (16, 17). The ENCHANTED study (8) also demonstrated that a lower dose can reduce the risk of intracranial hemorrhage. In this study, the low-dose rt-PA did not reduce the risk of bleeding, indicating the complexity of real-world scenarios. A possible explanation is that the low-dose group included a higher number of patients at greater risk of bleeding. Our results support physicians in making proactive dose selections during treatment.

In summary, this study has uncovered the current utilization of different doses of rt-PA in the treatment of AIS, as well as the factors that influence its use. Both patient and physician factors play significant roles in treatment decision-making, highlighting the complexity of these decisions. Physicians need to make choices based on a consideration of the patient’s specific circumstances, the potential risks and benefits of treatment, and their personal experience. Future research should focus on how to optimize the decision-making process to ensure that all patients receive the most appropriate treatment for their conditions. Additionally, enhancing physicians’ understanding and adherence to treatment guidelines, as well as their ability to assess patient-specific situations, is crucial for improving treatment outcomes.

### Limitations of study

This study has several limitations. First, as a single-center, retrospective, observational study, it may be subject to selection and information bias, potentially affecting the generalizability of the results. Second, although we adjusted for potential confounding factors for potential confounding factors, residual or unmeasured confounders may still influence the outcomes. Additionally, the absence of detailed analysis regarding physicians’ specific rationale for dose selection limits our understanding of decision-making motivations.

## Data Availability

The raw data supporting the conclusions of this article will be made available by the authors, without undue reservation.
